# Facile synthesis and characterization of nanorods Pb-MOF for toxic rodenticide detection

**DOI:** 10.1186/s13065-025-01579-y

**Published:** 2025-07-22

**Authors:** Ayman S. Eliwa, Magdi E.A. Zaki, Mostafa A. Besher, Gehad G. Mohamed

**Affiliations:** 1 Chemistry Department, Faculty of Science, Cairo University, Giza, 12613 Egypt; 2 Nanoscience Department, Basic and Applied Sciences Institute, Egypt-Japan University of Science and Technology, New Borg El Arab, Alexandria, 21934 Egypt; 3 Department of Chemistry, Faculty of Science, Imam Mohammad Ibn Saud Islamic University (IMSIU), Riyadh, 11623 Saudi Arabia

**Keywords:** Pb-MOF, Bromadiolone, Ultrasonic assisted method, Electrochemical sensor

## Abstract

One of the most powerful anticoagulant rodenticides is bromadiolone and its detection is essential because of its extensive application and possible environmental and health risks. Bromadiolone is a highly potent second-generation anticoagulant rodenticide that poses significant risks to both human health and the environment. Due to its high toxicity, even low levels of exposure can lead to severe poisoning in non-target species, including pets, wildlife, and humans. Furthermore, bromadiolone is known for its environmental persistence, accumulating in ecosystems and entering food chains. Therefore, the sensitive and accurate detection of bromadiolone residues is essential for monitoring contamination, ensuring public safety and guiding appropriate regulatory measures. Effective detection methods play a vital role in minimizing accidental poisoning, assessing environmental impact, and supporting forensic investigations related to toxic exposure incidents. This study presents the preparation and characterization of lead organic frameworks (Pb-MOFs) designed for the selective and sensitive detection of bromadiolone. Utilizing a combination of transition metals and organic linkers, we developed a series of MOFs with tailored pore sizes and functional groups to enhance their interaction with bromadiolone molecules. The synthesized MOFs were characterized through multiple techniques, including X-ray diffraction (XRD), scanning electron microscope (SEM), Fourier-Transform infrared spectroscopy (FT-IR), and Brunauer-Emmett-Teller (BET) surface area analysis, to verify their crystalline structure, morphology, functional groups, surface area, and pore size. The surface area was determined to be 1304.27 m^2^ g^− 1^ according to the BET analysis. With an average pore size of 4.61 nm, the calculated total pore volume was 2.13 cm^3^g^− 1^. An important step forward in chemical research is the utilization of the produced Pb-MOF in the electrochemical detection of the powerful anticoagulant rodenticide bromadiolone. We incorporated lead ions into the MOF structure to enhance its electrochemical sensitivity, enabling high detection of bromadiolone..

**Supplementary Information:**

The online version contains supplementary material available at 10.1186/s13065-025-01579-y.

## Introduction

**Fig. 1:**
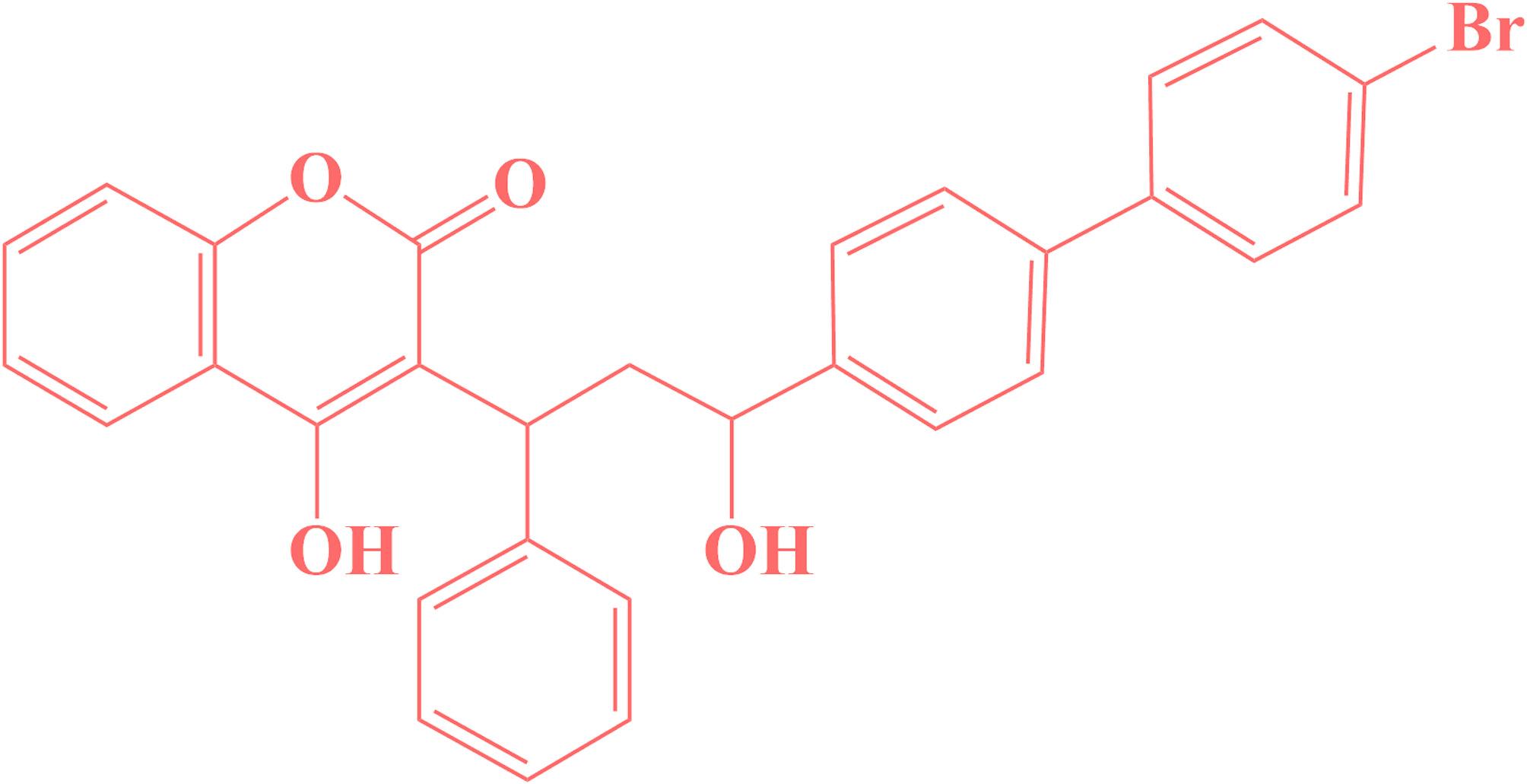
Chemical formula of bromadiolone

Bromadiolone is a powerful anticoagulant rodenticide and widely used for rodent reduction, particularly in rats and mice. It belongs to the second generation of 4-hydroxy-coumarin derivatives and functions as a vitamin K antagonist, disrupting the blood clotting process [1, 2]. This disruption occurs because bromadiolone inhibits the recycling of vitamin K, an essential component for the synthesis of clotting factors [3, 4]. As a result, affected animals suffer from internal bleeding, which can lead to death within a few days of ingestion. First introduced in the United States in 1980, bromadiolone has since become a critical tool in pest management due to its high efficacy and its formula shown in Fig. 1. In contrast to first-generation anticoagulants that need numerous doses for lethality [5–7], bromadiolone can be deadly upon a single consumption. This characteristic makes it particularly effective against rodent populations that have developed resistance to other anticoagulants. Bromadiolone is typically formulated as pellets or bait blocks containing 0.005% of the active ingredient. These products are often dyed blue-green or red to help identify exposure in non-target species. Due to its high toxicity, bromadiolone is primarily intended for professional use, with strict regulations to minimize accidental exposure to children, pets, and wildlife [7, 8]. It has a low potential for mobility in soil but can be more mobile in sandy soils. In aquatic environments, bromadiolone is highly toxic to fish and other aquatic organisms, although it is not typically applied near water bodies. Wildlife, particularly birds of prey, can be affected through secondary poisoning by consuming rodents that have been invested in bromadiolone. Several analytical methods have been employed for the determination of bromadiolone, including high-performance liquid chromatography (HPLC), gas chromatography-mass spectrometry (GC-MS), and spectrophotometry. While these techniques offer high sensitivity and selectivity, they often require complex sample preparation, expensive instrumentation, and longer analysis times. In contrast, the electrochemical method used in this study offers several advantages such as simplicity, rapid response, low cost, portability, and the potential for on-site detection. These characteristics make it particularly suitable for routine monitoring and field applications. Although electrochemical methods may have limitations in selectivity compared to chromatographic techniques, the use of appropriate electrode modifications and optimization can effectively address this issue. Therefore, the electrochemical approach was chosen for its practicality and efficiency in detecting bromadiolone. Electrochemical detection of bromadiolone is a valuable analytical technique due to its sensitivity, specificity, and cost-effectiveness. Bromadiolone detection is crucial for monitoring environmental contamination and ensuring food safety, as well as for forensic and clinical toxicology applications [9, 10]. Metal-organic frameworks (MOFs) are a class of crystalline materials composed of metal ions or clusters coordinated to organic ligands, forming [11–14] porous structures [15–17]. These frameworks have garnered significant attention because of their catalytic properties, such as high surface area, tunable pore sizes, and versatile chemical functionalities [18, 19]. These features give MOFs high suitability for various applications, including gas storage, catalysis, drug delivery, and particularly in the field of electrochemical sensors [12–13–20]. The use of metal-organic frameworks to modify electrodes for the electrochemical detection of bromadiolone represents a significant advancement in analytical chemistry [11–14–21–23]. MOFs are highly porous materials composed of metal ions or clusters coordinated to organic ligands, which provide a large surface area and tunable chemical functionalities [24–27]. These properties make MOFs ideal for enhancing the performance of electrochemical sensors [28–32]. MOFs can significantly improve the sensitivity and selectivity of electrochemical sensors for bromadiolone detection [21–33–36]. The high surface area of MOFs increases the number of active sites available for bromadiolone interaction, leading to enhanced signal strength. Additionally, the tunable nature of MOFs allows for the incorporation of specific functional groups that can selectively bind to bromadiolone molecules, reducing interference from other substances [6–10]. In practical applications, MOF-modified electrodes have been used to detect bromadiolone in environmental samples such as soil and water [37–39]. The process typically involves extracting bromadiolone from the sample matrix, followed by electrochemical analysis using the modified electrode. This approach allows for rapid and accurate detection of bromadiolone at low concentrations, which is essential for environmental monitoring and ensuring food safety.

## Experimental

### Chemicals

All compounds utilized in the studies were of purity p.a. unless specified differently. The 4-aminobenzoic acid and phthalaldehydic acid with purity 98% were procured from Sigma Aldrich. The amount of bromadiolone with purity 98% (Sigma-Aldrich) needed was dissolved in acetone with purity 98% (Scharlau) to create a 1 × 10^− 3^ M solution, which was then kept in a dark refrigerator at 4 ^o^C. Diluting using an acetate buffer of the appropriate pH value allowed for the daily acquisition of lower concentrations. Under the guidance of a pH meter, acetate buffer prepared by mixing of acetic acid and sodium acetate with purity 98% (Sigma-Aldrich). making the necessary pH adjustments with the help of NaOH and HCl.

### Instrumentation

Voltammetric measurements were taken using a potentiostat chi660e (CH Instruments, Inc.). A three-electrode configuration was used to carry out the experiment. Three electrodes were used: one for working current Gold electrode (GE) with diameter of 2 mm, one for reference current (Ag/AgCl) Electrode with diameter of 2 mm, and one auxiliary current of platinum wire. All three electrodes originated from Monokrystaly in the Czech Republic. pH was measured with a Fisher Scientific Accumet AB150 pH-meter and used an ultrasonic bath made by Schlalltec GmbH, Germany’s Bandelin Sonorex.

### Synthesis of schiff base ligand linker

**Fig. 2:**
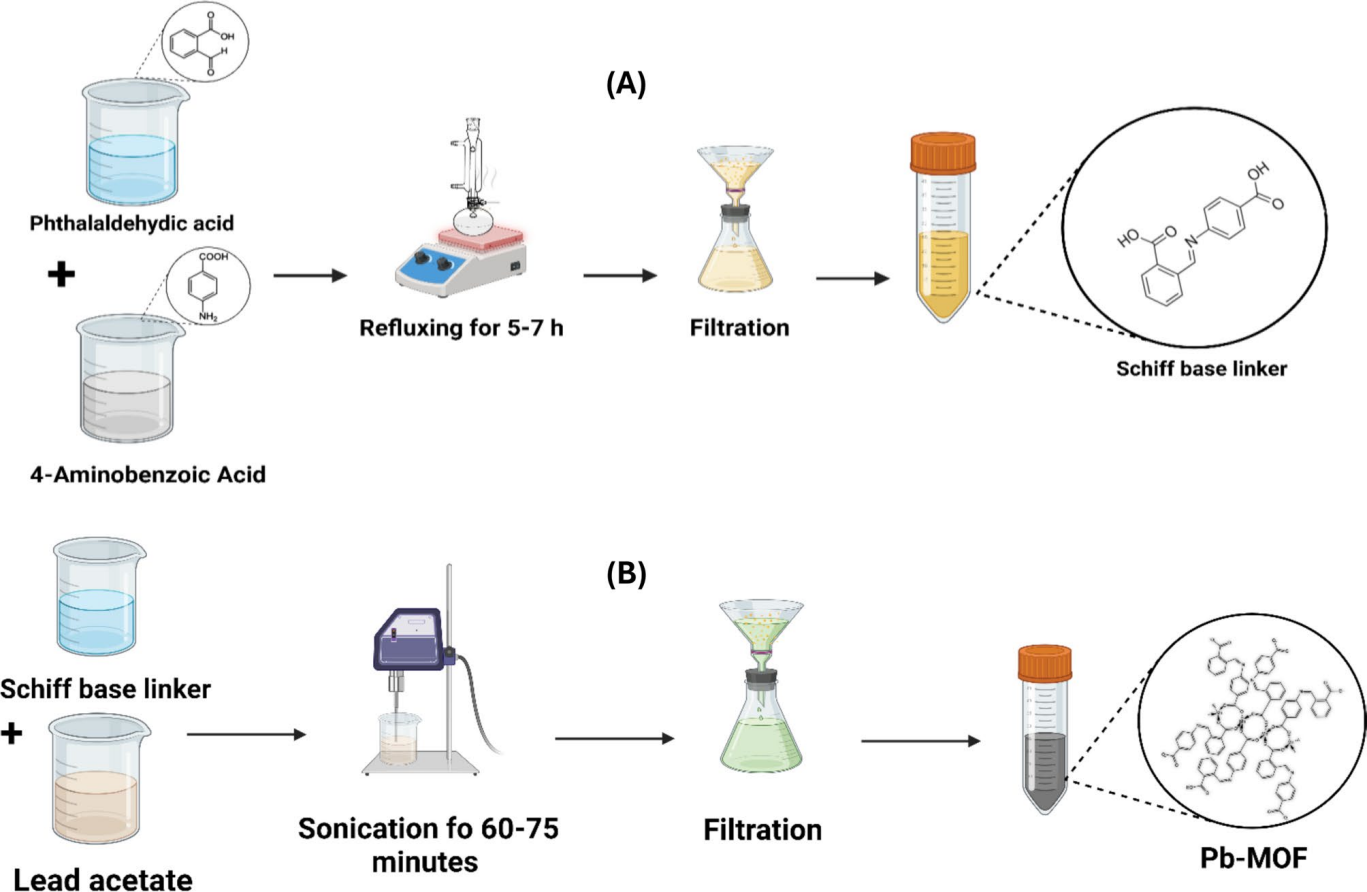
(**A**) Schiff base linker synthesis and (**B**) Pd-MOF ultrasonic preparation

The linker (H_2_L) was prepared by mixing an ethanolic solution of phthalaldehydic acid (5 g) with that of 4-aminobenzoic acid. In a ratio of 1:1 [40]. The amine was combined with the aldehydic solution and thereafter subjected to reflux for 5–7 h as shown in Fig. 2 (A). The solution was filtered, and the linker was successively rinsed with cold ethanol until it became clear. The solid linker was dried, achieving a yield of 87%.

### Synthesis of Pb-MOF

Fig. 2 (B) illustrated the process of ultrasonic synthesis of Pb–MOF. The H_2_L Schiff base ligand was dissolved, with a molar amount of 2 g and 5.4 mmol, in 50 mL of pure ethanol. [41]. It was mixed with 30 mL of ethanol to create a solution of lead acetate dihydrate (1.4 g, 3.7 mmol). Lead acetate dihydrate and H₂L have a molar ratio of 1:2. Then, placed them in a water-covered container. After that, the blend was sonicated for 60 to 75 min at 40 kHz frequency, taking one-second breaks between on and off periods. The trial kept the ultrasonic power at 60 watts all the time. Once the reaction time ended, centrifugation was used to separate the product. Fifty milliliters of water were used and ten milliliters of ethanol three times to thoroughly wash the precipitate. The last step was to dry the precipitate for 12 h at 130 °C. Afterwards, the product was let cool down naturally at room temperature.

### Modified gold electrode (MGE) preparation

A mixture of 0.03 g of Pb-MOF and 0.2 mL of ethanol was sonicated for 45 min to create the Pb-MOF/GE. Afterward, immerse the GE in the solution and allow it to dry at room temperature.

## Results and discussion

### Schiff base ligand characterization (H_2_L)

**Fig. 3:**
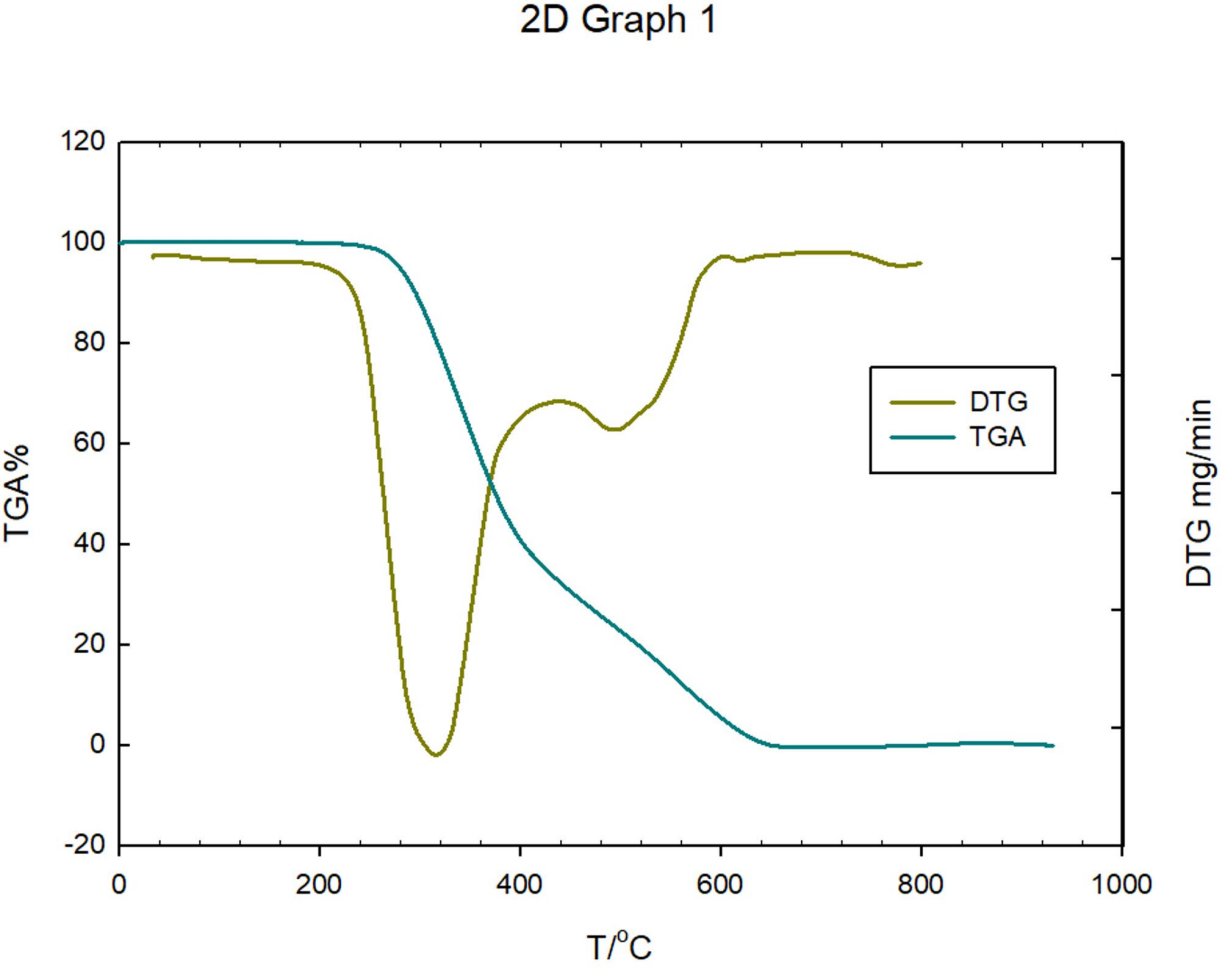
Thermal analysis of Schiff base ligand

A linker was synthesized from a pre-existing ligand to aid the production of MOFs. To synthesize the ligand, 2-((4-carboxyphenyl)imino)methyl) benzoic acid (H_2_L) was formed by the condensation of phthalaldehydic acid and 4-aminobenzoic acid. The white color linker characterized using an uncomplicated approach. The elemental analyses revealed that the calculated values of C: 67.10%, N: 5.36%, and H: 4.20% were close to the found values (C: 67.10%, N: 5.36%, and H: 4.20%). The findings coincided with those computed for the proposed formula (C_15_H_11_NO_4_), exhibiting high purity as a result of its sharp melting point at 270 ºC. The FT-IR spectrum of the free ligand (H_2_L) showed the disappearance of the amine group band of 4-aminobenzoic acid at 1601 cm^− 1^, accompanied by the emergence of a new band for ν(CH = N) azomethine at 1630 cm^− 1^. The stretching bands of ν_asym_(COO^−^) and ν_sym_(COO^−^) were observed at 1468 cm^− 1^ and 1321 cm^− 1^, respectively [42]. The ligand’s ^1^H-NMR spectra indicate at 5.8 ppm aromatic proton signals attribution, carboxylic proton group signals at 12.3 ppm, and singlet proton group multiplet signals in the 6.5–7.9 ppm range. The mass spectrum of the investigated Schiff base ligand was primarily occupied by molecular ion peaks with intensities varying from moderate to high. The ligand molecule C_15_H_11_NO_4_, which has a molecular mass of 269.25 g/mol, corresponds to a clear parent peak at m/z = 269.07 amu in the Schiff base ligand’s mass spectrum. The thermal analysis of the ligand (H_2_L) is shown in Fig. 3, which revealed its degradation process using differential thermogravimetric analysis (DTG) and thermogravimetric analysis (TGA). There were two stages to this breakdown, and it was completed at 620 °C.

### Pb-MOF characterization

#### FT-IR analysis

**Fig. 4:**
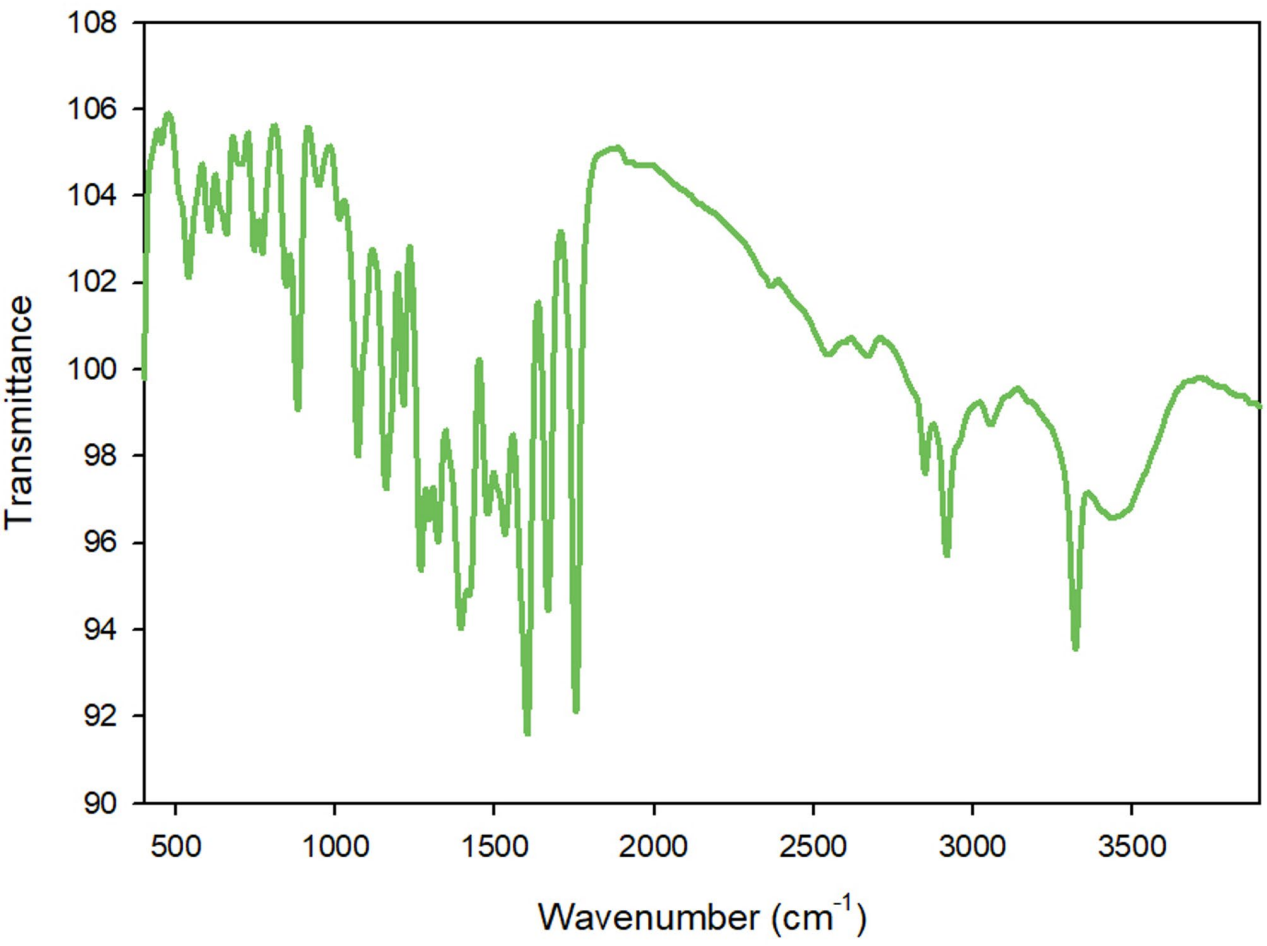
The synthesized Pb-MOF FT-IR spectrum

Firstly, because of O-H vibrations of water molecules, a strong absorption band was found between 3435 and 2918 cm^− 1^ where data presented in Fig. 4 showed this. The Pb-MOF’s Fourier-Transform Infrared (FT-IR) spectrum showed strong bands at 1394 and 1602 cm^− 1^. These bands are caused by the different stretching modes (symmetric and asymmetric) of the coordinated (–COO) group, respectively. Based on the finding, it looks like the carboxyl group (-COOH) of H_2_L is involved in how it binds to lead ions. It is possible for the carboxylate group to chelate in the Pb-MOF and this showed that the carboxylate parts of the lead MOF work together in a chelating bidentate way. At 539 cm^− 1^, a Pb-O bond peak appeared, corresponding to its existence in the metal-organic framework that was being studied.

#### Powder X-ray diffraction pattern (PXRD)

**Fig. 5:**
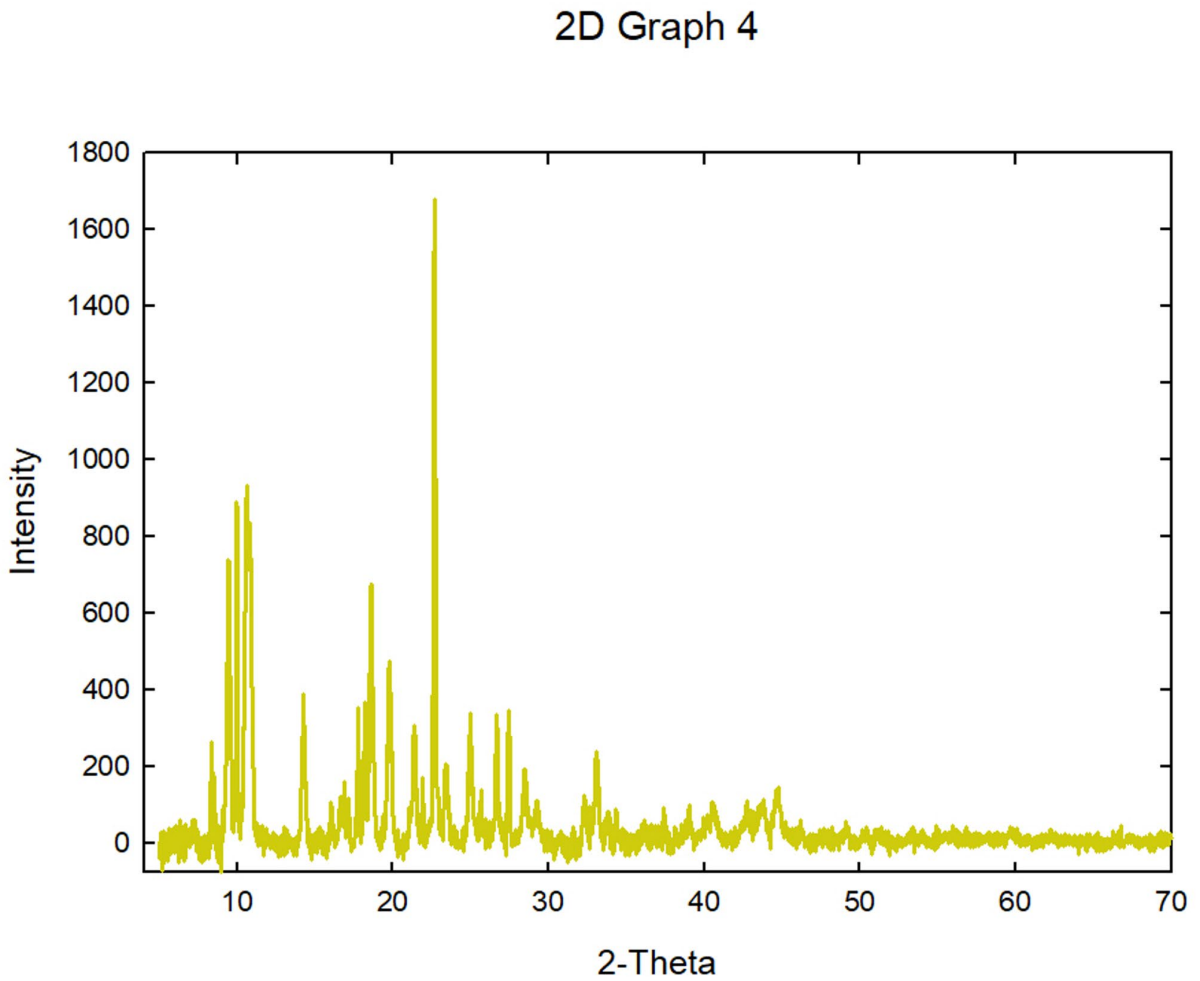
The synthesized Pb-MOF PXRD diffractogram

Many properties can be well elucidated using the powser X-ray diffraction (PXRD) method. Fig. 5 showed that the synthesized Pb-MOF had a very structurally crystalline X-ray powder diffraction pattern. In the Pb-MOF material, diffraction peaks were detected at 9.9^o^, 10.6^o^, 10.8^o^, 14.2^o^, 18.6^o^, 19.8^o^, 22.6^o^, 26.6^o^, and 27.4^o^. In Fig. 5, previous research has declared that the (PXRD) pattern of (Pb-MOF) is similar to the one found in this study [42, 43].

#### BET surface area analysis

**Fig. 6:**
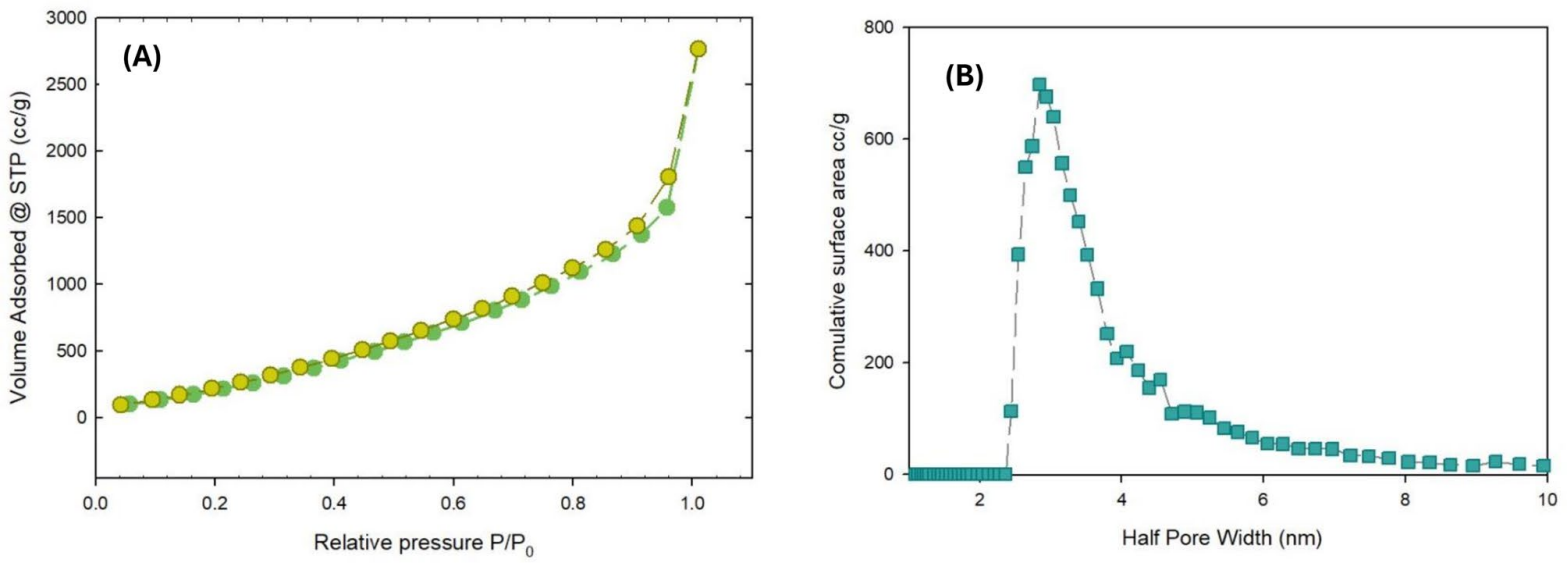
(**A**) Pb-MOF adsorption-desorption isotherm and (**B**) pore size distribution curve

Using N_2_ adsorption, volumetric measurements were used to determine the fabricated Pb-MOF’s porosity and surface area. Standard N_2_ adsorption-desorption studies were performed on Pb-MOF at 77 K. The findings are shown in Fig. 6 (A) and (B). The type IV isotherm is attributed to the synthesized Pb-MOF, which is common for nanomaterials. The surface area was determined to be 1304.27 m^2^ g^− 1^ with a pore size of 4.61 nm associated with a total pore volume of 2.13 cm^3^ g^− 1^.

#### SEM image of Pb-MOF

**Fig. 7:**
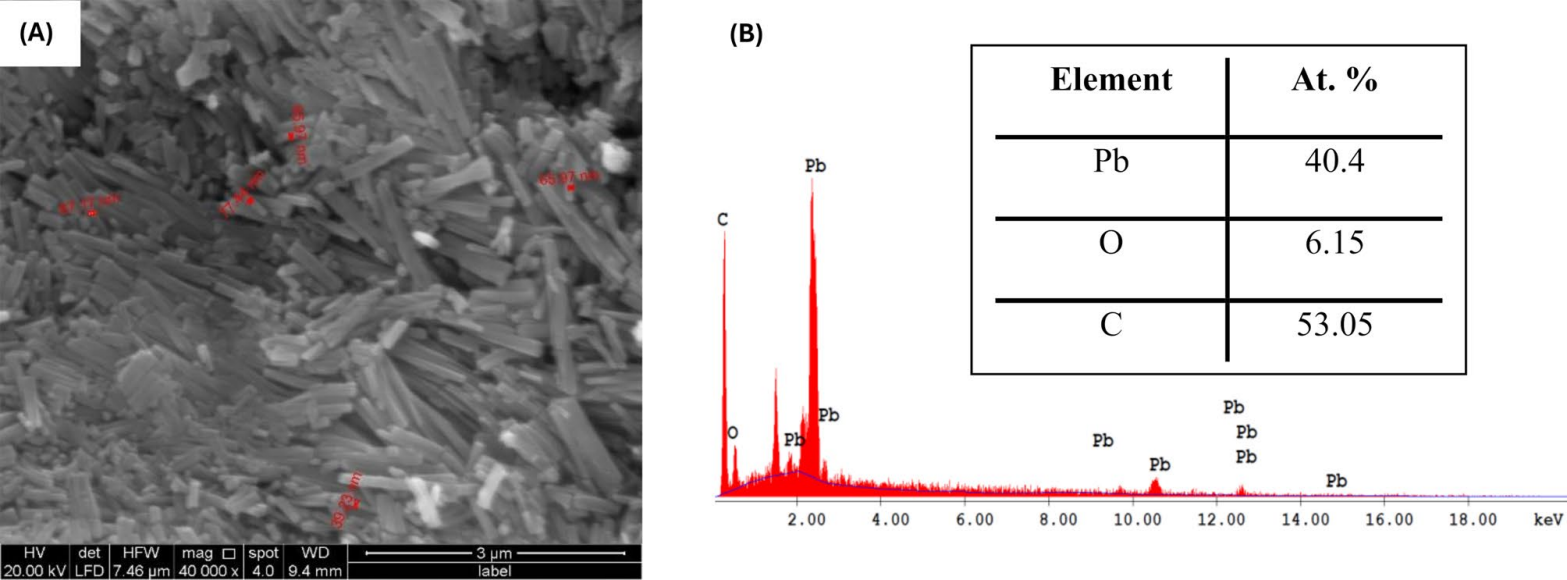
(**A**) SEM images of synthesized Pb-MOF and (**B**) Pb-MOF EDX spectrum

The size, form, and structure of Pb-MOFs that were sonochemically synthesized were studied using scanning electron microscope. It proved that ultrasonic irradiation creates smaller particles than the solvothermal synthesis method [44]. The synthesized Pb-MOF is shown in Fig. 7 (A) in the form of scanning electron micrographs. The scanning electron micrograph demonstrated the successful production of sheets-shaped nanoparticles within the 39.23–77.44 nm range.

#### EDX analysis

Using the important EDX analysis method lets you figure out what the parts are made of. Fig. 7 (B) showed Pb-MOF EDX spectrum, which showed that lead, carbon and oxygen were all present. The order in which the Pb-MOF’s parts were found and their percentages: C > Pb > O. Also, EDX defined parts showed that the Pb-based MOF was successfully synthesized. These parts would work as active sites on the surface of the adsorbent.

#### Thermogravimetric analysis

**Fig. 8:**
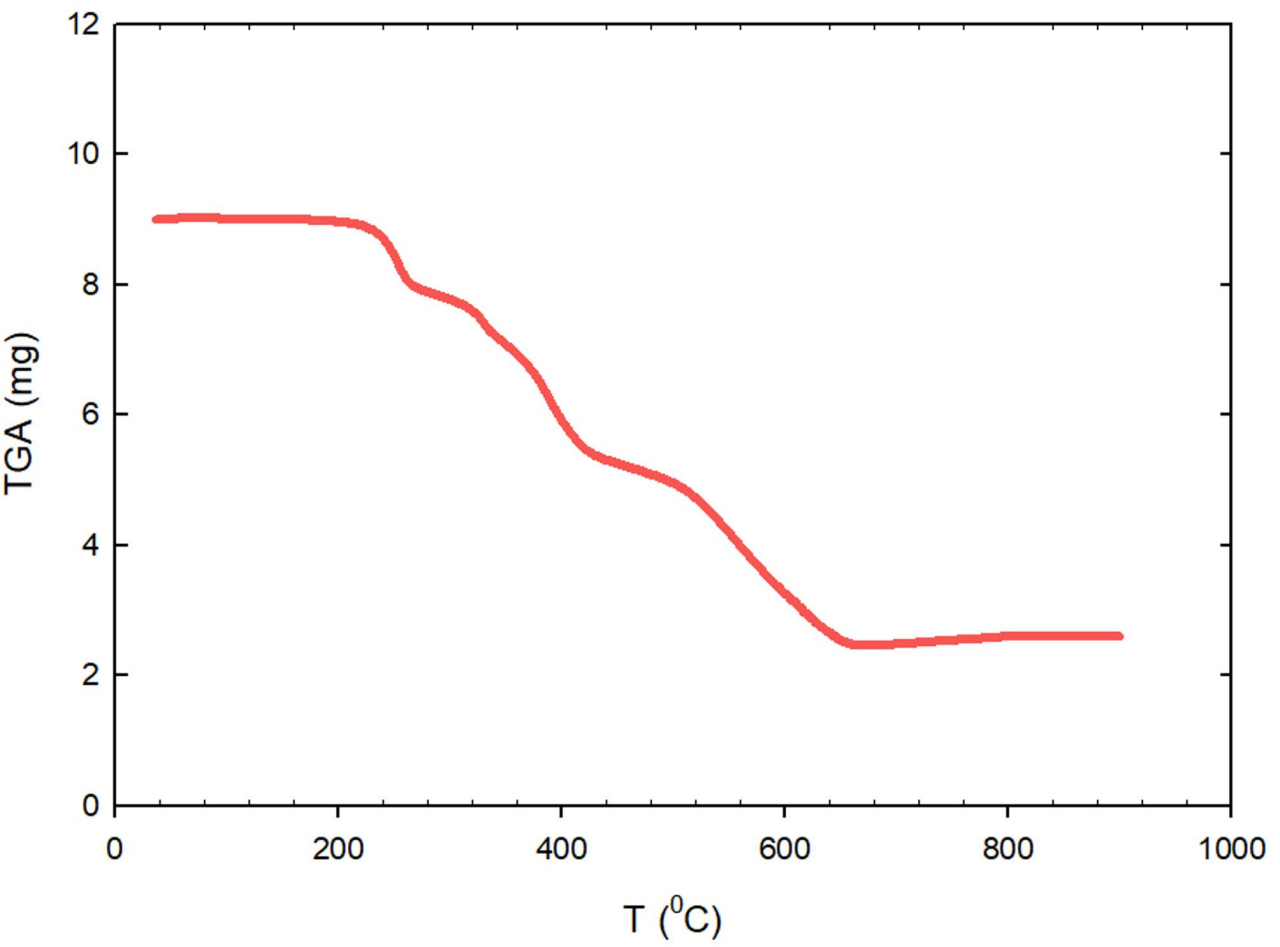
Pb-MOF TGA curve

Thermogravimetric analysis (TGA) was used to look into how the Pb-MOF compound broke down when heated. Fig. 8 showed that the compound went through three important temperature changes. In the first stage, about 13.146% of the weight loss is due to getting rid of the remaining water molecules and ethanol liquid in the pores, as well as the remaining unreacted carbon materials. At temperatures between 25 and 290 °C, this happened through volatilization. In the next phase, which happened between 290 and 340 °C, the mass continues to drop by about 8.192% over time. Starting with this step, coordinated water molecules are taken out of the Pb-MOF. This created new active sites. In the third stage, within the temperature range of 340.95–623.04 °C, the Pb-MOF sample exhibited a weight reduction of about 54.3%.

## Voltammetric behavior of bromadiolone

**Fig. 9:**
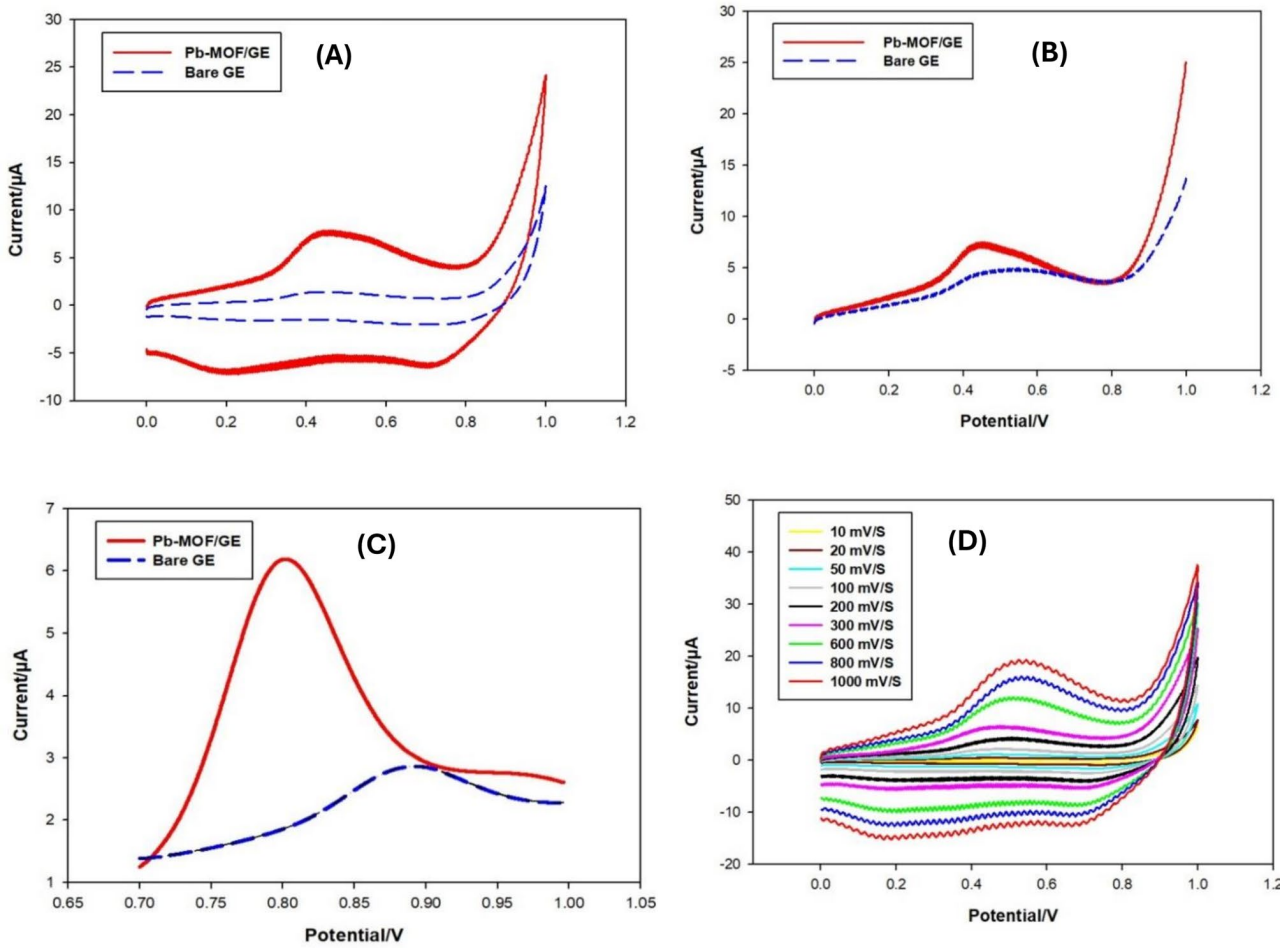
(**A**) CV of bare GE and Pb-MOF/GE for bromadiolone in acetate buffer (pH = 4.0), (**B**) Linear sweep voltammograms (LSV) of bare GE and Pb-MOF/GE for bromadiolone in acetate buffer (pH = 4.0), (**C**) Differential pulse voltammograms (DPV) of bare GE and Pb-MOF/GE for bromadiolone in acetate buffer (pH = 4.0) and (**D**). Cyclic voltammograms of bromadiolone in acetate buffer (pH = 4.0) at different scan rates

Measurements using voltammetry prior to the first test, GE was activated electrochemically using cyclic voltammetry (CV) as shown in Fig. 9 (A). The process is made up of 30 cycles that take place in an electrolyte with a starting potential of 0 V and a switching potential of + 1.1 V. The voltage-dependent relation studied using CV for bromadiolone on a Pb-MOF/GE was, and the relationship between pH and scan rate (ʋ) was found. Using ʋ = 300 mV/s, cyclic voltammograms were made from E_in_ 0 V to E_switch_ +1.1 mV. This value was changed between 10 and 1000 mV/s to see what effect it had. Linear sweep voltammetry (LSV) was measured using the following optimized LSV parameters: Input voltage = 0 V, output voltage = + 1.0 V, eigenvalue = 5 mV/s, quiet time = 2s, and sensitivity = 10^− 5^ as shown in Fig. 9 (B). The acetate buffer with a pH of 4.0 was chosen for the bromadiolone analysis using differential pulse voltammetry (DPV). These were the optimized DPV parameters: the input potential is + 0.7 V, the output potential is + 1.0 V, the frequency is 50 mV/s, the pulse height is + 60 mV, and the width of the pulse is 16 ms as shown in Fig. 9 (C).

### Effect of scan rate

It was found that bromadiolone’s voltammetric behavior changed with scan rate (ʋ) between 10 and 1000 mV/s. It was seen that as the scan rate went up, the anodic peak current (Ipa) and cathodic peak current (Ipc) both went up. The anodic peak moved to a more positive value, and the cathodic peak moved to a more negative value. This is shown in Fig. 9 (D).

### Effect of pH

**Fig. 10:**
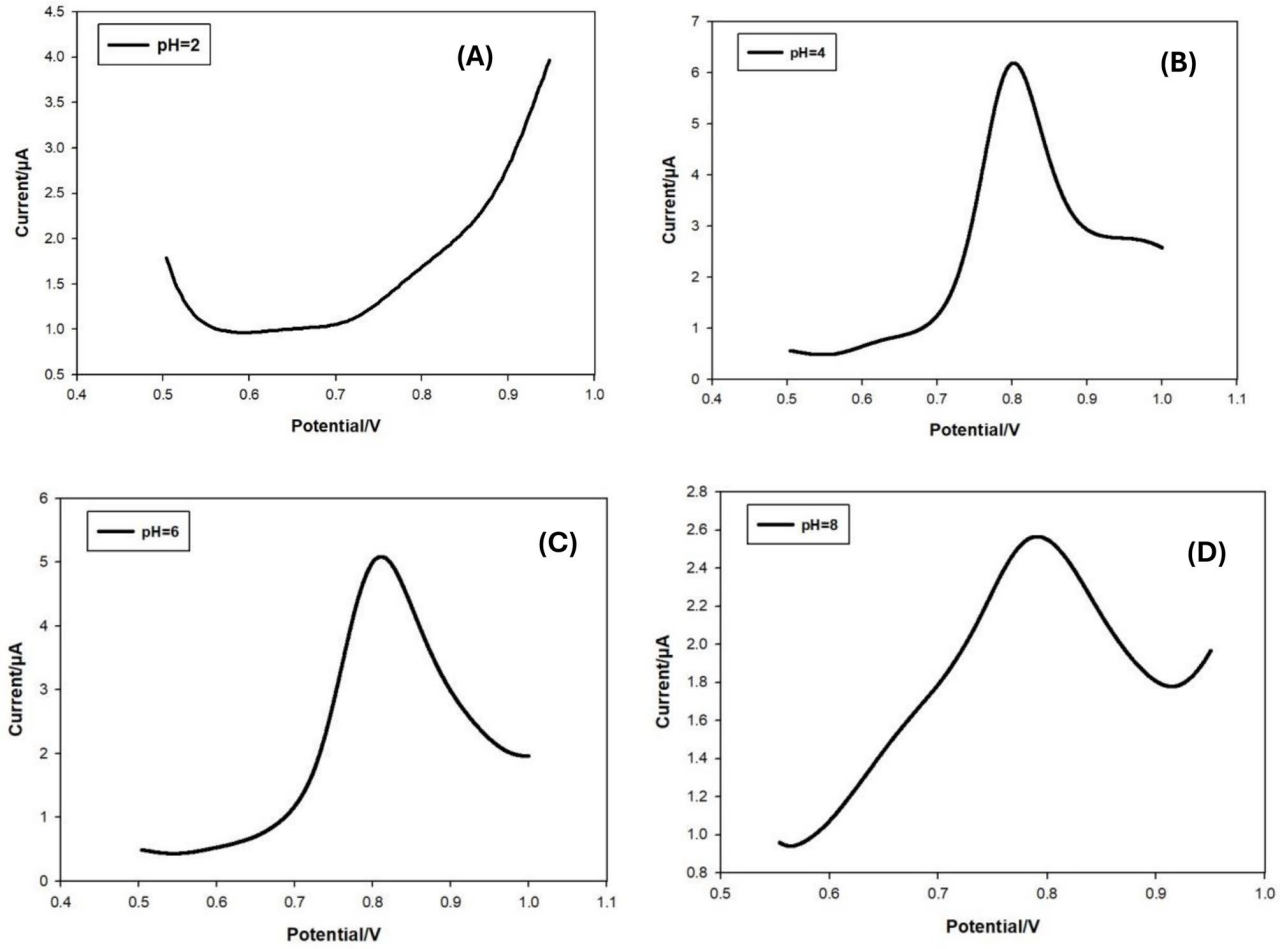
Differential pulse voltammograms (DPV) of 1×10^-4^ M bromadiolone at different pH

The first tests looked at how the voltammetric behavior of bromadiolone changed depending on the pH of the solution that was used. The LSV in Fig. 9 (B) showed that bromadiolone gave one oxidation peak at a potential of + 0.81 V in a buffer of acetate with a pH of 4.0. Also, no matching reduction peaks were seen. This meant that the electrode reaction could not be undone. The form and location of the bromadiolone peaks are changed by pH, as shown in Fig. 10 (A, B, C, D). The strongest and most reliable signs were seen at pH of 4.0. Consequently, this medium was employed for all the next studies.

### Effect of time

**Fig. 11:**
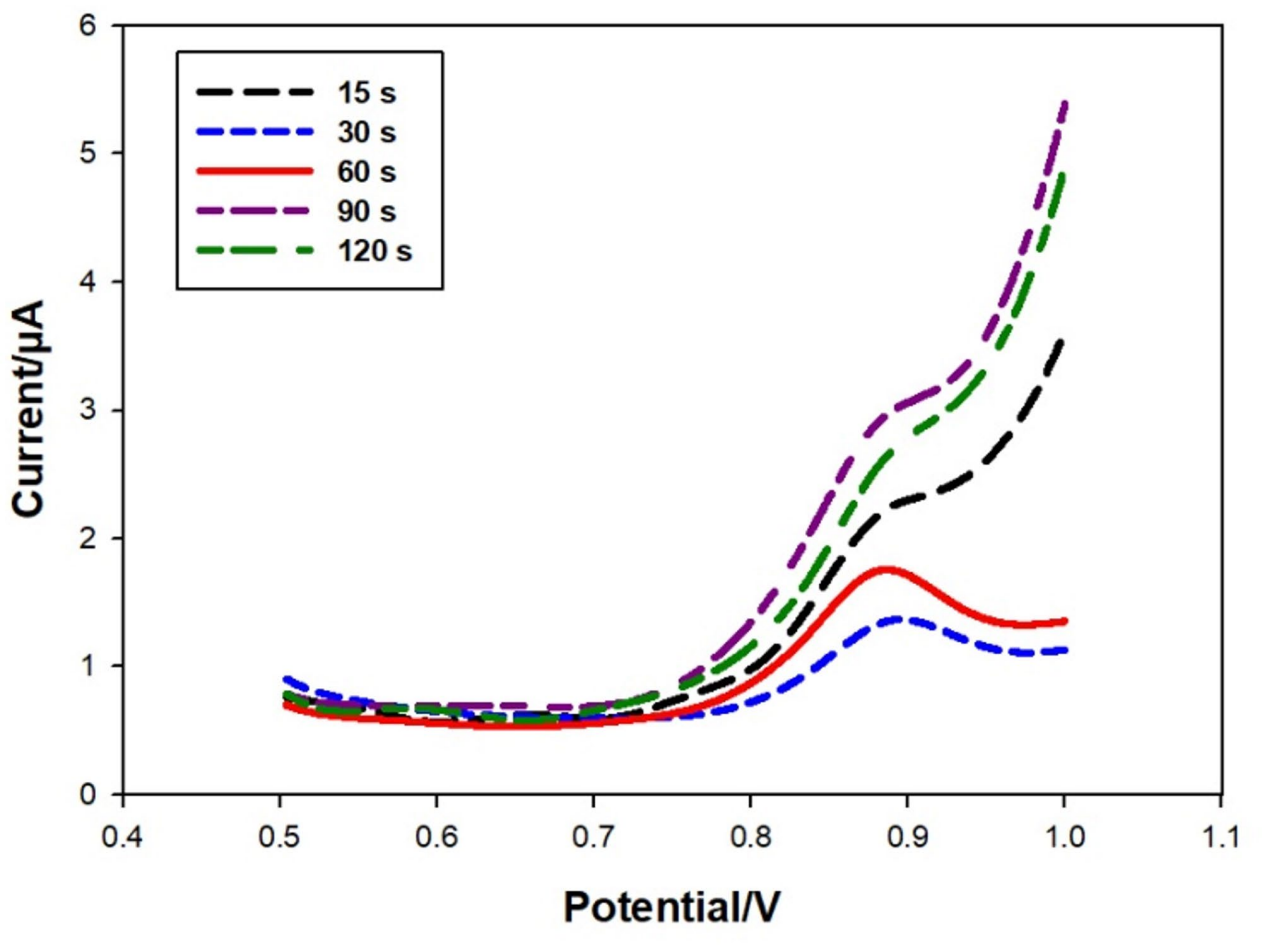
Differential pulse voltammograms (DPV) at different intervals

The modified electrode’s adsorption of bromadiolone attenuated the sensor signal, and the adsorption duration (waiting time) was examined prior to analysis (15–120 s). The peak current augmented with prolonged adsorption time, attaining its maximum at 60 s. Fig. 11 illustrated this. As time goes on, the peak current decreases. This means that after 60 s, the bromadiolone has fully attached to the Pb-MOF/GE surface. The modifier’s high porosity results in rapid attainment of saturation. Consequently, a waiting period of 60 s was selected for the subsequent trial.

### Limit of detection and calibration curve

**Fig. 12:**
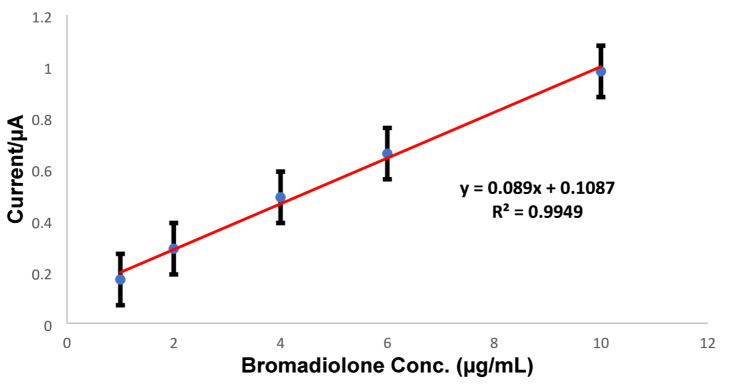
Calibration plot of peak current (Ip) on Pb-MOF/GE for different bromadiolone concentrations (1 - 10 µg/mL) in 0.2 M acetate buffer solution (pH = 4)

The response of the Pb-MOF/GE to different doses of bromadiolone was documented under optimum circumstances. The limit of detection (LOD) was determined as 3.3 times the standard deviation of the intercept divided by the slope, while the limit of quantification (LOQ) was estimated as 10 times the standard deviation of the intercept divided by the slope. The limit of detection (LOD) and the limit of quantitation (LOQ) were determined to be 1.7 and 5.1 µg/mL, respectively. The calibration curve was derived using the bromadiolone concentration and the peak current (Ip). Fig. 12 illustrated a broad linear range from 1 to 10 µg/mL (R² = 0.9949) with the mathematical relationship y = 0.089x + 0.1087.

## Conclusion

In this study, a lead based metal-organic framework (Pb-MOF) was successfully synthesized and characterized and specifically designed for the detection of bromadiolone, a widely used anticoagulant rodenticide. The structural and functional properties of this MOF was meticulously analyzed, demonstrating their high surface area, porosity, and the presence of functional groups conducive to bromadiolone interaction. Our findings indicated that this lead-based MOF exhibited excellent sensitivity and selectivity towards bromadiolone. The reusability tests confirmed the robustness and stability of the MOFs over multiple detection cycles, making them viable for practical applications. These results suggested that lead-based MOF are promising candidates for environmental monitoring and public health protection against bromadiolone contamination. Future research will focus on optimizing the synthesis process to enhance the performance of these MOFs further and exploring their applicability in real-world environmental samples. The development of portable detection devices incorporating these MOFs could revolutionize the field of rodenticide detection, providing a reliable and efficient tool for safeguarding environmental and human health.

## Electronic supplementary material

Below is the link to the electronic supplementary material.

Supplementary Material 1

## Data Availability

The datasets used and/or analysed during the current study are available from the corresponding author on reasonable request.
