# Supplementary material for: Facile synthesis and characterization of nanorods Pb-MOF for toxic rodenticide detection
Source: BMC Chem. 2025 Jul 22;19(1):219. doi: 10.1186/s13065-025-01579-y (PMC12285016; doi:10.1186/s13065-025-01579-y)
Supplement: Supplementary file 1 — Supplementary Material 1 [file 13065_2025_1579_MOESM1_ESM.docx]

**Instruments**

The FT-IR spectra obtained using a Perkin-Elmer 1650 spectrometer (4000-400 cm^-1^) as KBr pellets were used in this study. TMS was used as an internal standard and ^1^H-NMR spectra were recorded as a solution in DMSO-*d_6_* on a 300 MHz Varian-Oxford Mercury at room temperature (RT). The mass spectra were obtained using the EI technique at 70 eV on a Hewlett-Packard MS-5988 GS-MS instrument. Carbon, hydrogen, and nitrogen microanalyses were performed at Cairo University Microanalytical Center utilizing a CHNS-932 (LECO) Vario Elemental Analyzer. TG and DTG were performed using a Shimadzu TG-50H thermal analyzer at a rate of 5 ^o^C/min from ambient temperature to 800 °C. A SEM Model Quanta 250 FEG (Field Emission Gun) linked to an EDX unit (Energy Dispersive X-ray Analyses) was used to capture an image of the MOF, with an accelerating voltage of 30 K.V., magnification of 14 up to 1000000, and resolution for Gun (National Research Center, Egypt). The X-ray diffraction (XRD) was recorded in Egypt Nanotechnology Center (EGNC) using a Bruker D8 Discover (Bruker AXS Inc., 35 KV, 30 mA) X-ray diffractometer with a step size of 0.02 and speed scan of 0.016 utilizing Cu K radiation (= 1.5406) for 2h with 2 varying between 5 and 50. Gas adsorption tests using N_2_ as the adsorptive gas at 77 K were used to determine the BET surface area and pore size distribution. Prior to the adsorption tests, the materials were evacuated for 4-12 hours under high vacuum. The Brunauer-Emmett-Teller (BET) theory was used to calculate the analysis, which was done with a Nova Touch LX2 analyzer. UV-Vis spectra were recorded using a Shimadzu UVmini-1240 UV-Vis spectrophotometer. Attension-Theta of Biolin Scientific assessed the contact angle at Egypt Nanotechnology Center, Cairo University, El-Sheikh Zayed, Egypt.
